# Immune-responsiveness of CD4^+^ T cells during *Streptococcus suis* serotype 2 infection

**DOI:** 10.1038/srep38061

**Published:** 2016-12-01

**Authors:** Marie-Pier Lecours, Corinne Letendre, Damian Clarke, Paul Lemire, Tristan Galbas, Marie-Odile Benoit-Biancamano, Jacques Thibodeau, Marcelo Gottschalk, Mariela Segura

**Affiliations:** 1 Laboratory of Immunology, Faculty of veterinary medicine, University of Montreal, St-Hyacinthe, Quebec, Canada; 2 Laboratory of Streptococcus suis, Faculty of veterinary medicine, University of Montreal, St-Hyacinthe, Quebec, Canada; 3 Laboratory of Molecular Immunology, Department of Microbiology, Infectiology and Immunology, University of Montreal, Montreal, Quebec, Canada; 4 Pathology Division, University of Montreal, 3200 rue Sicotte, St-Hyacinthe, J2S 2M2 Canada.

## Abstract

The pathogenesis of *Streptococcus suis* infection, a major swine and human pathogen, is only partially understood and knowledge on the host adaptive immune response is critically scarce. Yet, *S. suis* virulence factors, particularly its capsular polysaccharide (CPS), enable this bacterium to modulate dendritic cell (DC) functions and potentially impair the immune response. This study aimed to evaluate modulation of T cell activation during *S. suis* infection and the role of DCs in this response. *S. suis-*stimulated total mouse splenocytes readily produced TNF-α, IL-6, IFN-γ, CCL3, CXCL9, and IL-10. *Ex vivo* and *in vivo* analyses revealed the involvement of CD4^+^ T cells and a Th1 response. Nevertheless, during *S. suis* infection, levels of the Th1-derived cytokines TNF-α and IFN-γ were very low. A transient splenic depletion of CD4^+^ T cells and a poor memory response were also observed. Moreover, CD4^+^ T cells secreted IL-10 and failed to up-regulate optimal levels of CD40L and CD69 in coculture with DCs. The CPS hampered release of several T cell-derived cytokines *in vitro*. Finally, a correlation was established between severe clinical signs of *S. suis* disease and impaired antibody responses. Altogether, these results suggest *S. suis* interferes with the adaptive immune response.

*Streptococcus suis* is an important swine pathogen primarily associated with meningitis, albeit other systemic pathologies have been described^1^. *S. suis* is considered an emerging pathogen and represents a threat to human health, especially in Asia. Indeed, *S. suis* is the principal cause of adult meningitis in Vietnam, the second in Thailand, and the third in Hong Kong^1^. Furthermore, in the last years important human outbreaks of *S. suis* streptococcal toxic shock-like syndrome (STSLS) occurred in Asia with fatality rates nearby 20%^1^. Among 35 serotypes that have been described, serotype 2 is the most virulent for both pigs and humans, and most of the studies have been performed with this serotype. The capsular polysaccharide (CPS), which defines the serotype, is considered a major virulence factor of *S. suis* serotype 2^2^.

Dendritic cells (DCs) are potent antigen-presenting cells and are critical for bridging innate and adaptive immune responses^3^. DCs capture and process invading pathogens to present their antigens to corresponding lymphocytes. Following antigen uptake, DCs increase the expression of different cell surface molecules (known as maturation process) and the release of cytokines. After DC migration to draining lymph nodes, co-stimulatory molecules bind to naive T cells, leading to T cell activation^3^. The production of cytokines, such as interleukin (IL)-12, by mature DCs provides additional signals for the acquisition of T cell effector functions^4^.

CD4^+^ T cells are important for the development of immunity to bacterial infections. After interaction with their cognate antigen presented by activated DCs, naive CD4^+^ T cells proliferate and polarize towards different CD4^+^ lineages, which then shape the immune response. The best characterized CD4^+^ lineages are T helper type 1 (Th1), which drives the immune response mainly against intracellular pathogens; Th2, which promotes humoral responses; Th17, which contributes to the elimination of extracellular pathogens; and various regulatory T cell (Treg) populations, which prevent the development of autoimmunity^5^. However, there is accumulating evidence that the CD4^+^ T cell lineages are not as stable as initially thought. Substantial heterogeneity and plasticity, as assessed by cytokine production patterns, have been observed within these subsets, particularly when generated *in vivo* and during an infection^5^. Hence, it seems more likely that multiple polarized CD4^+^ T cell subsets are generated. These effector cells secrete large quantities of cytokines and chemokines^6^. For example, the Th1 cells secrete great amounts of IFN-γ, TNF-α, and IL-2 whereas the Th2 cells secrete high levels of IL-4, IL-5, IL-9, and IL-13^6^.

Despite the increasing number of studies, the pathogenesis of *S. suis* infection is still not completely understood and, to date, attempts to control the infection are hampered by the lack of an effective vaccine^7^. Mouse bone marrow-derived DCs have been shown to be an effective model to study the immune response of the host during *S. suis* infection^8^,^9^. There is evidence that mouse DCs are activated after *S. suis* infection, as suggested by the up-regulation of the co-stimulatory molecules CD40 and CD86 as well as cytokine and chemokine production, including TNF-α, IL-1β, IL-6, IL-12p70, and IL-23^8^,^9^. However, *S. suis* possesses virulence factors able to modulate the functions of DCs, mainly production of cytokines and opsono-phagocytosis, possibly lessening the immune response^8^,^9^. In fact, we and others have shown that the presence of CPS on *S. suis* strongly reduces DC activation/maturation and *S. suis* internalization, and/or modulates the IL-10/IL-12 and IL-10/TNF-α cytokine production in favor of a more anti-inflammatory profile by either human-, mouse- or swine-derived DCs^8^,^10^,^11^. Here, we test the hypothesis that encapsulated *S. suis* affects the development of T cell-dependent immune responses, which might represent one of the consequences of *S. suis* modulation of DC functions. Indeed, this work addresses for the first time the role of CD4^+^ T cells in the host adaptive immune response against *S. suis* and the potential contribution of the bacterial CPS to the modulation of this response.

## Results

### Dose-dependent role of CD4^+^ T cells in survival after *S. suis* infection

**Figure 1:**
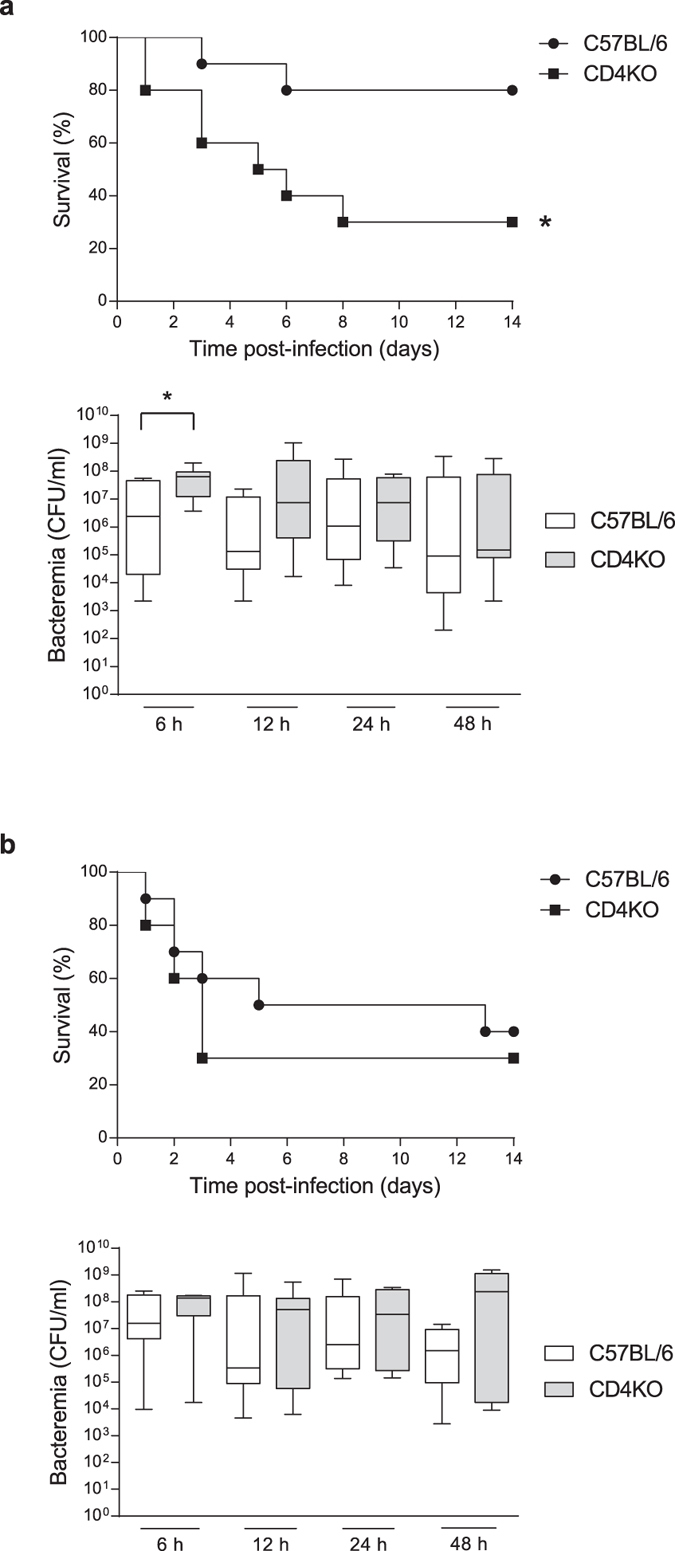
CD4KO are more susceptible to *S. suis* infection than control C57BL/6 mice at a low infectious dose. Mice (n = 10 per group × 2 individual experimental infections) were infected intraperitoneally with (**a**) 1 × 10^7^ CFU or (**b**) 5 × 10^7^ CFU of *S. suis* wild-type strain P1/7. Survival levels were recorded and systemic bacteremia levels of infected mice was monitored at 6, 12, 24, and 48 h after infection. Blood was drawn by tail puncture and serially diluted in PBS prior to plating on blood agar dishes. Individual colonies were counted and data expressed as CFU/mL of blood. *P < 0.05, indicates statistically significant difference compared to control mice.

CD4 knockout (KO) and control C57BL/6 mice were infected with *S. suis* wild-type (WT) strain P1/7 (1 × 10^7^ CFU) in a preliminary investigation of the role of CD4^+^ T cells during *S. suis* infection. Mice devoid of functional CD4^+^ T cells died significantly more rapidly than control mice 14 days after infection (Fig. 1a). The same result was obtained at 21 days after infection (data not shown). CD4KO mice also showed higher levels of bacteremia at 6 h after infection than control mice. However, there was no statistical difference in bacteremia between both groups at later time points (Fig. 1a). Upon infection with a higher infectious dose, CD4KO mice showed similar survival curves and bacteremia levels to those observed in control mice, both after 14 days (Fig. 1b) and 21 days (data not shown). These results suggest that CD4^+^ T cells play only a transient and limited role in the control of *S. suis* infection.

### Encapsulated *S. suis* induces a type-1 pro-inflammatory environment in the spleen during systemic infection

**Figure 2:**
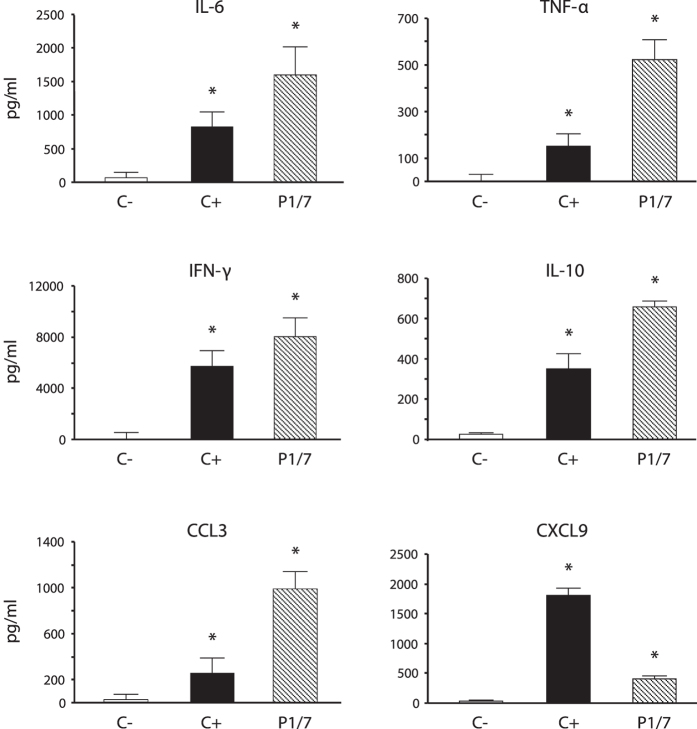
Total splenocytes produce *ex vivo* type-1 pro-inflammatory cytokines in response to *S. suis* systemic infection. Mice were infected intraperitoneally with a dose of 5 × 10^7^ CFU of *S. suis* wild-type strain P1/7 (n = 3 per group × 3 individual experimental infections). Spleens were harvested 6 h post-infection and total splenocytes plated at 5 × 10^6^ cells/well. After 6 h of incubation, gentamycin was added to the culture to prevent cell toxicity. Cells were then incubated for 48 h and supernatants were collected for cytokine analysis by ELISA. Non-stimulated cells from mock-infected animals served as negative control for basal expression (C−). Cells stimulated with Concanavalin A (0.1 μg/ml) were used as positive control (C+). Data are expressed as mean ± SEM (in pg/ml) from 3 different experimental infections. *P < 0.05 denotes values that are significantly higher than those obtained with splenocytes from non-infected mice (C−).

In order to characterize the immunological environment during *S. suis* systemic infection, an *ex vivo* approach was first used to measure the production of different cytokines in the spleen. Total splenocytes from mice infected with *S. suis* WT strain P1/7 were collected and incubated in cell culture plates for 48 h. Splenocytes from control (placebo) animals were used as negative controls and ConA-treated splenocytes were used as positive controls. Under these conditions, a significant production of IL-6 and TNF-α as well as high levels of IFN-γ were observed, indicating the development of a type-1 pro-inflammatory response (Fig. 2). Infected splenocytes also released significant amounts of IL-10, suggesting a role of this regulatory cytokine in maintaining homeostasis during the inflammatory process. Interestingly, the presence of CCL3 and CXCL9, important chemokines involved in T cell recruitment, was observed (Fig. 2). The production of IL-4 was not detected (data not shown).

### CD4^+^ T cells are involved in the host immune response induced during *S. suis* infection

**Figure 3:**
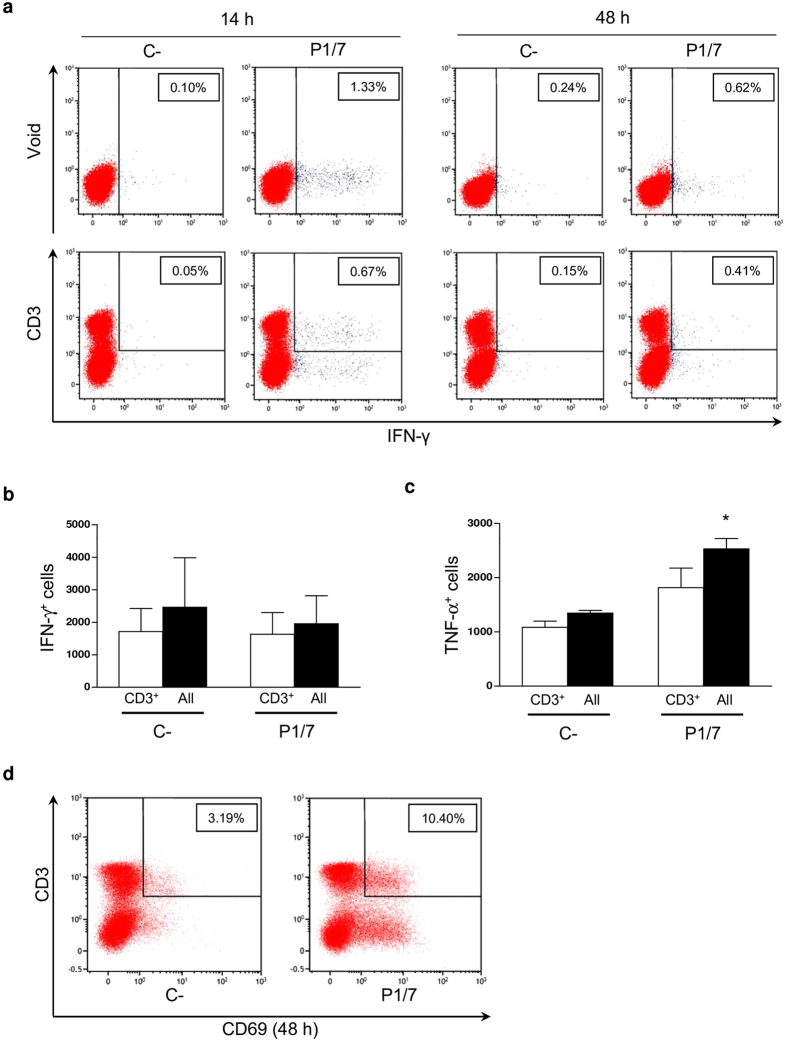
CD3^+^ T cells produce low *ex vivo* levels of IFN-γ and show poor activation in response to *S. suis* systemic infection. Mice were infected intraperitoneally with a dose of 5 × 10^7^ CFU of *S. suis* wild-type strain P1/7 (n = 3 per group × 3 individual experimental infections). Spleens were harvested 6 h post-infection and total splenocytes plated at 5 × 10^6^ cells/well. After 6 h of incubation, gentamycin was added to the culture to prevent cell toxicity. Non-stimulated cells from mock-infected animals served as negative control for basal expression (C−). Total splenocytes were incubated for 14 h or 48 h with Brefeldin A (3 μg/ml) added during the last 5 h of incubation. Cells were harvested and intracellularly stained for (**a**,**b**) IFN-γ, (**c**) TNF-α or surface stained for (**d**) CD69 in combination with several surface markers for multi-parametric FACS analysis. (**a**,**d**) Representative data from 3 different experimental infections based on CD3^+^ population or total splenic population (Void). (**b**,**c**) Number of either IFN-γ^+^ or TNF-α^+^ cells within the CD3^+^ population or within total splenic population (All) at 48 h. Data are expressed as mean ± SEM from 3 different experimental infections. FACS was performed using a FACSCanto II instrument. Fifty thousand gated events were acquired per sample and data analysis was performed using FACSDiva™ software. Fluorescence Minus One (FMO) control staining was performed for proper analysis and gating of target cells. *P < 0.05, indicates statistically significant difference compared to negative control cells (C−).

CD4^+^ T cells are key players in the development of host immune responses; however, their activation status and cytokine profile in response to *S. suis* infection has never been investigated. Firstly, we performed a multi-parametric flow cytometry (FACS) analysis of *ex vivo* total splenocyte production of IFN-γ, a Th1 signature cytokine. As shown in Fig. 3a, IFN-γ production was overall weak and hardly detectable by intracellular (IC)-FACS within the whole spleen cell population. CD3^+^ T cells contributed to ~50% of the IFN-γ response in the spleen of infected mice. However, when considering inter- and intra-experiment variations, this weak IFN-γ production was not statistically significant compared to control mice (Fig. 3b). Natural killer T (NKT) cells (NK1.1^+^CD3^+^) produced low to negligible levels of IFN-γ. Indeed, natural killer (NK) cells (NK1.1^+^) were the major contributors to IFN-γ production within the CD3^−^ population early during infection (M. Segura, unpublished observations). As expected, CD19^+^ cells (B cells) did not produce significant levels of this cytokine (data not shown). Similar findings were obtained when analyzing TNF-α production (Fig. 3c). Nevertheless, a strong expression of the early leukocyte activation marker CD69 was observed in total splenocytes from infected animals compared to control mice. Around 12.8% ± 2.5% (mean ± SEM) of cells expressed this marker within the CD3^+^ population, suggesting that a small portion of T cells have been activated during infection (Fig. 3d).

**Figure 4:**
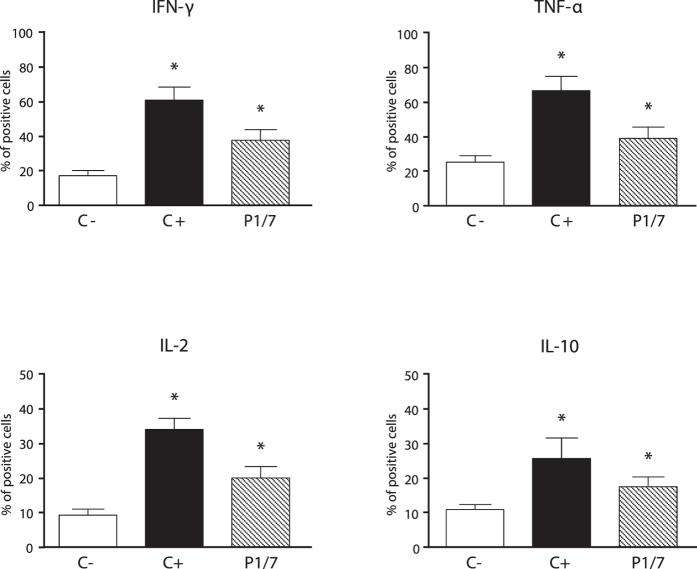
CD4^+^ T cells produce low *ex vivo* levels of IFN-γ, TNF-α, and IL-2 in response to *S. suis* systemic infection. Mice were infected intraperitoneally with a dose of 5 × 10^7^ CFU of *S. suis* wild-type strain P1/7 (n = 3 per group × 3 individual experimental infections). Spleens were harvested 6 h post-infection and total splenocytes plated at 5 × 10^6^ cells/well. After 6 h of incubation, gentamycin was added to the culture to prevent cell toxicity. Non-stimulated cells from mock-infected animals served as negative control for basal expression (C−). Cells stimulated with Concanavalin A (0.1 μg/ml) were used as positive control (C+). Total splenocytes were incubated for 48 h. Brefeldin A (3 μg/ml) was added during the last 5 h of incubation and CD4^+^ T cells were MACS-isolated from the culture, stained intracellularly for different cytokines and analyzed by FACS. Data are expressed as mean ± SEM (in % of positive cells) from 3 individual experimental infections. FACS was performed using a FACSCalibur instrument. Twenty thousand gated events were acquired per sample and data analysis was performed using CellQuest software. *P < 0.05, indicates statistically significant difference compared to negative control cells (C−).

As the frequency of activated CD3^+^ T cells during *S. suis* infection was very low and to better evaluate the role of CD3^+^CD4^+^ T cells, these target cells were MACS-isolated from *ex vivo* total splenocyte cultures and analyzed by IC-FACS. As shown in Fig. 4, CD4^+^ T cells contributed to the release of low, still significant levels of IFN-γ, TNF-α, and IL-2. These data suggest that CD4^+^ T cells indeed differentiate into Th1 cells, although low % of activated cells are observed relatively to positive control ConA-treated cells. Interestingly, a significant % of CD4^+^IL-10^+^ cells was also observed in *ex vivo* total splenocyte cultures (Fig. 4).

**Figure 5:**
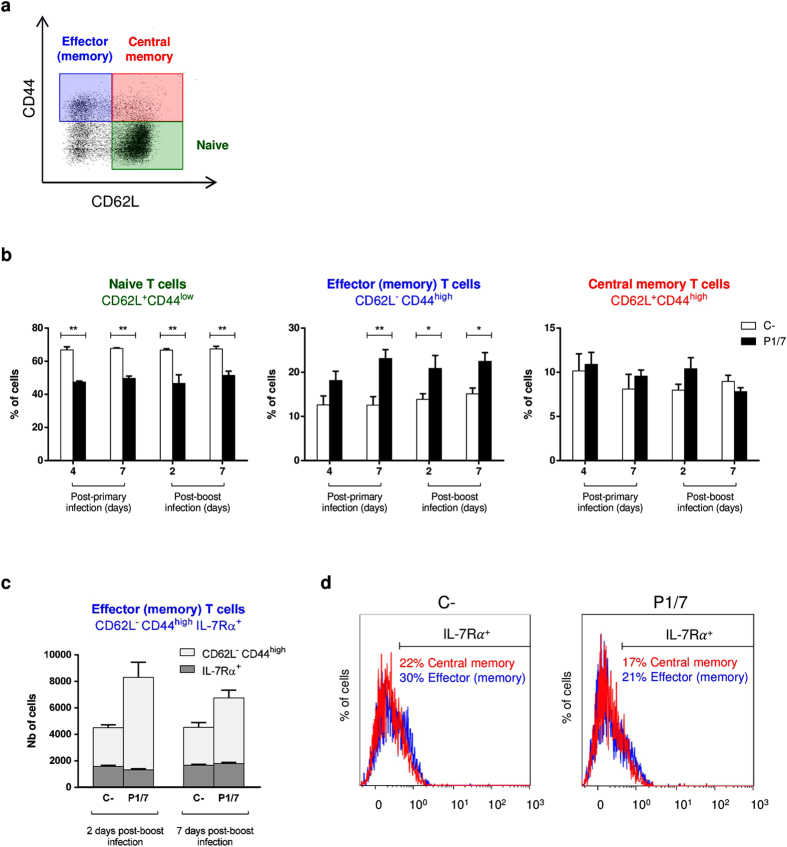
*S. suis* induces no expansion of the effector memory and central memory CD4^+^ T cell splenic subsets during primary and secondary infections. Mice were infected intraperitoneally with a dose of 1 × 10^7^ CFU of *S. suis* wild-type strain P1/7. Surviving animals who had previously displayed clinical symptoms were boosted with a second dose of 1 × 10^7^ CFU of *S. suis* wild-type strain P1/7 two weeks after initial infection. Spleens of animals with clinical symptoms and positive bacteremia were harvested 96 h post-primary infection or 48 h post-boost infection (n = 3 per group × 2 individual experimental infections). Total splenocytes were stained and analyzed by multi-parametric FACS. (**a**,**b**) Cells were gated on CD3^+^CD4^+^ cells, followed by gating CD62L^+^CD44^low^ (naive T cells), CD62L^−^CD44^high^ (effector [memory] T cells) and CD62L^+^CD44^high^ (central memory T cells). (**c**) A fifth surface marker, IL-7Rα^+^, was used to further identify memory cells (CD44^high^IL-7Rα^+^) within the effector (memory) subset. Data from the late time points were selected for the figure. (**d**) IL-7Rα^+^ cells reflect memory cells within the effector (memory) subset (blue histogram) and central memory subset (red histogram). Histograms from representative non-infected and infected mice 2 days post-boost infection were selected for the figure. (**b**,**c**) Data are expressed as mean ± SEM. FACS was performed using a FACSCantoII instrument. Thirty thousand events gated on CD3^+^CD4^+^ cells were acquired per sample and data analysis was performed using Kaluza® Flow Analysis software. Quadrants were drawn based on PE-Cy7- and BV421-control stains and were plotted on logarithmic scales. *P < 0.05 and **P < 0.01, indicate statistically significant difference compared to non-infected mice.

With the aim of measuring the frequency and level of activation of CD4^+^ T cells *in vivo* during infection, mice were injected i.p. with Brefeldin A solution and CD4^+^ T cells were directly isolated from the spleen 96 h post-primary infection. The % of CD4^+^IFN-γ^+^ cells was very low after a primary infection and not significantly different from controls. On the other hand, *in vivo* production of TNF-α and IL-2 by CD4^+^ T cells was significantly higher than control cells (see Supplementary Fig. S1). Similarly to *ex vivo* data, a significant production of IL-10 by CD4^+^ cells was also observed *in vivo* during *S. suis* primary infection. Surviving mice were challenged with a second infectious dose 2 weeks after primary infection and similarly treated with Brefeldin A. When CD4^+^ T cells were isolated 48 h post-boost, % of IFN-γ^+^, TNF-α^+^, IL-2^+^, and IL-10^+^ CD4^+^ cells were similar, or only slightly higher, than those observed 96 h post-primary infection (see Supplementary Fig. S1). This is consistent with the poor memory CD4^+^ T cell response that was observed in the spleen both after a primary infection (4 and 7 days) and secondary infection (2 and 7 days). In fact, while % of naive CD4^+^ T cells (CD62L^+^CD44^low^) showed a significant reduction at all time points after infection (Fig. 5b), % of memory CD4^+^ T cells (CD44^high^IL-7Rα^+^) remained unchanged when compared to naive mice (see Supplementary Fig. S2). More precisely, although the effector (memory) subset (CD62L^−^CD44^high^) showed a significant and sustained augmentation starting 7 days after a primary infection (Fig. 5b), this increase could not be attributed to the presence of actual memory cells among total spleen cells, as evidenced by the unchanged % in IL-7Rα^+^ cells within this subset (Fig. 5c and d, blue population and histograms). The lack of augmentation of this subset in the spleen could also be explained by cellular migration from primary lymphoid tissues to the periphery. However, the fact that the central memory subset (CD62L^+^CD44^high^) also remained unchanged and showed no significant increase in % of IL-7Rα^+^ cells, even after a secondary infection, is in agreement with the development of a poor memory CD4^+^ T cell response during *S. suis* infection (Fig. 5b and d, red population and histograms).

Finally, in agreement with a limited activation of CD4^+^ T cells, *S. suis* induced a weak specific antibody response. In addition, the generation of antibodies against the bystander antigen ovalbumin (OVA) was impaired in *S. suis*-infected animals. Interestingly, the anti-OVA antibody production was significantly lower in infected animals displaying severe clinical signs than in infected animals showing milder clinical signs (see Supplementary Results and Fig. S4, S5, and S6).

### *S. suis* CPS impairs cytokine release by T cells

**Figure 6:**
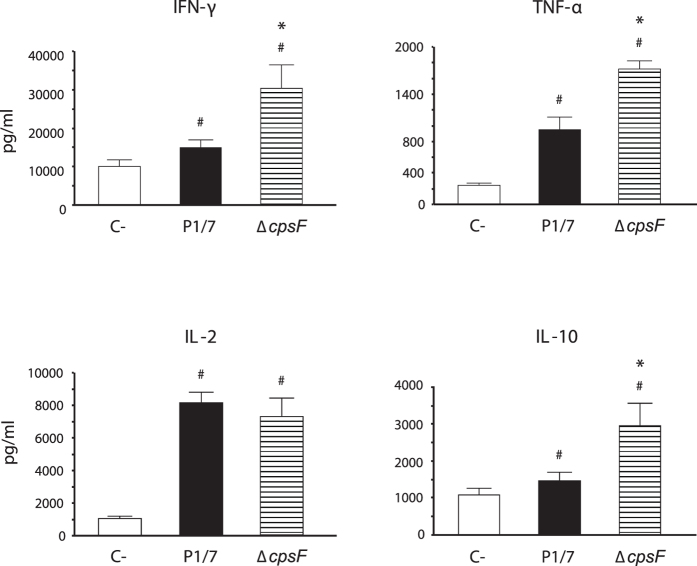
*S. suis* capsular polysaccharide interferes with cytokine production by CD4^+^ T cells *in vitro*. Bone marrow-derived dendritic cells (bmDCs) were infected with either *S. suis* wild-type strain P1/7 or its non-encapsulated isogenic mutant Δ*cpsF* (MOI: 1) for 1 h. Extracellular bacteria were killed by antibiotic treatment and cultures washed prior to addition of freshly isolated splenic CD4^+^ T cells from naive mice (T cell: DC ratio of 5: 1). Cocultures were incubated for 48 h, resuspended in fresh medium containing 10 ng/ml of IL-2 for 72 h (resting period) and then transferred to anti-CD3 coated plates for 48 h. Supernatants were then collected and cytokines quantified by ELISA. Non-stimulated cocultures served as negative controls (C−) for basal expression. Data are expressed as mean ± SEM (in pg/ml) from 5 different experiments. ^#^*P* < 0.05, indicates statistically significant differences compared to negative controls (C−). *P < 0.05, indicates statistically significant differences between cocultures infected with the wild-type strain P1/7 and those infected with the non-encapsulated mutant Δ*cpsF*.

Previous experiments showed that albeit CD4^+^ cells are involved in the immune response induced during *S. suis* infection, their activation status seems to be compromised and their role in controlling infection limited. To better characterize the effect of *S. suis* on CD4^+^ T cell functions, we performed *in vitro* DC-T cell cocultures. As *S. suis* is a well-encapsulated bacterium, we also evaluated the impact of CPS on the activation of CD4^+^ T cells *in vitro*, using a non-encapsulated mutant strain. Supernatants from coculture experiments were collected and tested by ELISA for the presence of CD4^+^ T cell-derived cytokines. No significant cytokine production was observed in single cell cultures (DCs or T cells alone) that served as controls (data not shown). The WT strain P1/7 induced the release of low but significant levels of IFN-γ and TNF-α. Low levels of IL-10 released by CD4^+^ T cells were also observed in response to WT *S. suis* activation *in vitro* (Fig. 6). In contrast, the non-encapsulated mutant strain Δ*cpsF* induced significantly higher levels of TNF-α, IFN-γ, and IL-10 by CD4^+^ T cells. Compared to other cytokines, *S. suis*-activated CD4^+^ T cells released large amounts of IL-2 and this production was not modified in cocultures stimulated with the Δ*cpsF* mutant strain (Fig. 6). These results suggest that the presence of the CPS on *S. suis* modulates CD4^+^ T cell activation without affecting autocrine IL-2 secretion.

### *S. suis* interferes with T cell expression of co-stimulatory molecules

**Figure 7:**
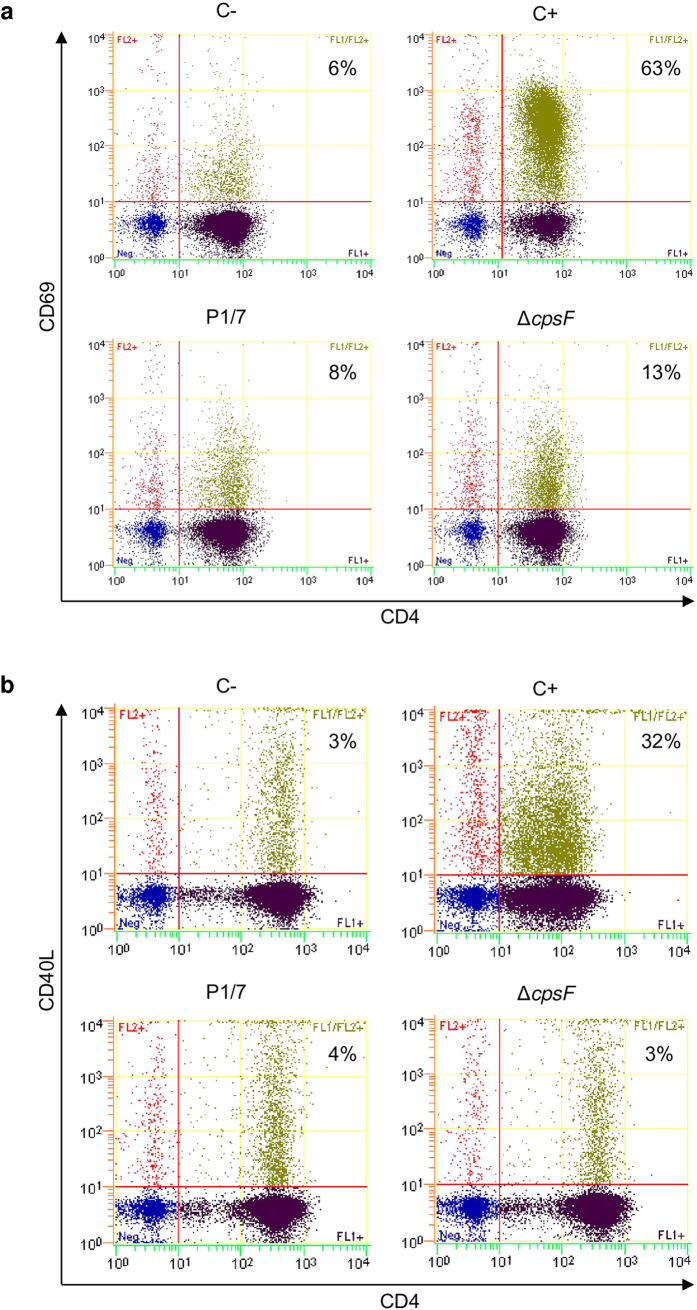
*S. suis* modulates CD4^+^ T cell surface expression of CD69 and CD40L *in vitro*. Bone marrow-derived dendritic cells (bmDCs) were infected with either *S. suis* wild-type strain P1/7 or its non-encapsulated isogenic mutant Δ*cpsF* (MOI: 1) for 1 h. Extracellular bacteria were killed by antibiotic treatment and cultures washed prior to addition of freshly isolated splenic CD4^+^ T cells from naive mice (T cell: DC ratio of 5: 1). Cocultures were incubated for 8 h, cells harvested and (**a**) CD69 or (**b**) CD40L expression analyzed by FACS. Cocultures incubated with medium alone served as negative controls (C−). Cocultures treated with either Concanavalin (0.1 μg/ml) or phorbol myristate acetate (15 ng/ml) + ionomycine (150 ng/ml) served as positive controls (C+) for CD69 and CD40L expression, respectively. FACS was performed using a Cell Lab Quanta^TM^ SC MPL MultiPlate Loader instrument. Twenty thousand gated events were acquired per sample and data analysis was performed using Cell Lab Quanta Collection/Analysis software. Quadrants were drawn based on FITC- and PE-control stains and were plotted on logarithmic scales. Representative data from 3 different experiments. Numbers in the upper quadrants indicate the % of CD4^+^CD69^+^ or CD4^+^CD40L^+^ cells.

In addition to cytokine production, expression of surface molecules on CD4^+^ T cells is an essential event for proper T cell activation. To measure the ability of *S. suis* to induce optimal activation of CD4^+^ T cells, we measured surface expression of CD69 and CD40L. Figure 7 shows that *S. suis* failed to induce a significant increase in surface expression of these molecules *in vitro*, compared to uninfected control cells. Cocultures infected with the non-encapsulated mutant strain Δ*cpsF* showed a low increase in CD69 expression by T cells (Fig. 7a); however levels of CD40L expression remained unchanged (Fig. 7b). Similar results were observed independently of the incubation time (3, 8, 24, and 48 h of coculture incubation, not shown).

### Reduced numbers of splenic CD4^+^ T cells after *S. suis* infection

**Figure 8:**
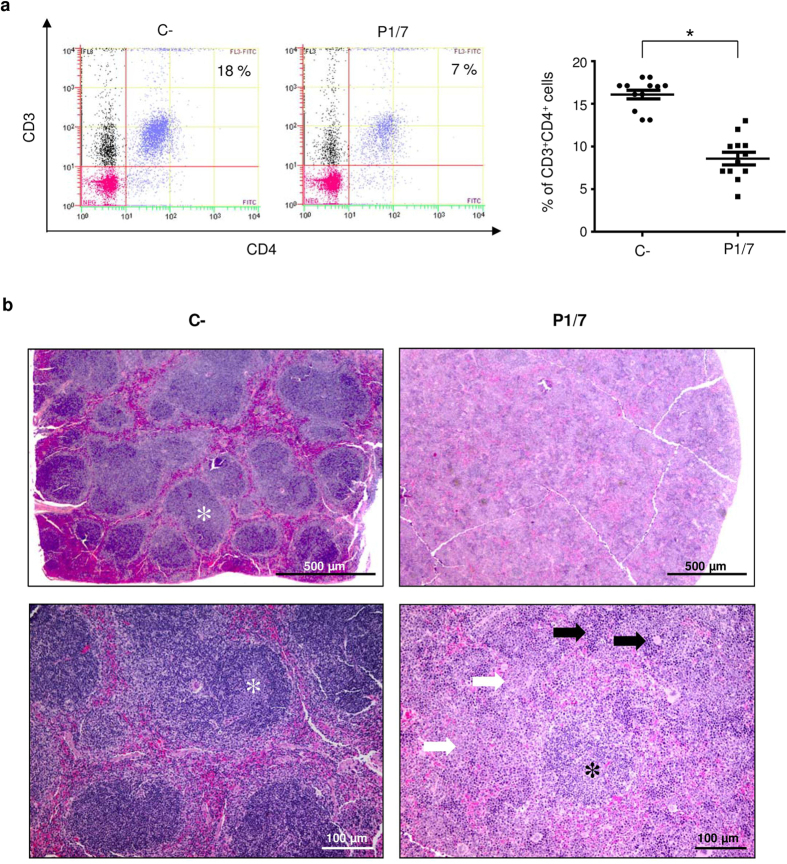
Spleens from *S. suis*-infected mice show reduced numbers of CD4^+^ T cells concurrently with histopathological changes, as compared to control mice. Mice were infected intraperitoneally with a dose of 1 × 10^7^ CFU of *S. suis* wild-type strain P1/7. Non-infected control animals were also included. (**a**) At 8 days post-primary infection, % of CD3^+^CD4^+^ T cells in the spleens of infected and control animals were evaluated by FACS. Representative data (*left*) and individual mouse data (*right*) are presented from 3 experimental infections (n = 12). FACS was performed using a Cell Lab Quanta^TM^ SC MPL MultiPlate Loader instrument. Twenty thousand gated events were acquired per sample and data analysis was performed using Cell Lab Quanta Collection/Analysis software. Quadrants were drawn based on FITC- and PE-Cy5-control stains and were plotted on logarithmic scales. (**b**) In parallel to FACS analysis, half-spleens were preserved in formalin for histopathological analysis. Spleen sections from infected animals were compared to controls and the level of lymphoid depletion (lymphatic nodules – asterisks), granulopoiesis (white arrows), and extramedullary hematopoiesis (black arrows) was evaluated on a 4-grade scale (grade 4 being the most severe). 2.5X images (top panel) show a reduction in the number and cellularity of lymphatic nodules. 10X images (bottom panel) show an increase in extramedullary hematopoiesis as well as in granulopoiesis.

To better understand the dynamics of T cell activation *in vivo*, total numbers of splenic CD4^+^ and CD8^+^ T cells were quantified during the *S. suis* infection. Spleens from control and infected mice were collected 2, 4, 6, and 8 days post-primary infection and 2, 4, 6, and 8 days post-boost infection. Similar % of CD4^+^ T cells were observed between infected and non-infected controls during the first 6 d post-primary infection (data not shown). However, a significant decrease in the numbers of these cells was observed at 8 days post-primary infection (Fig. 8a). This reduction in the number of CD4^+^ T cells persisted after challenge infection and gradually came back to normal by 6 to 8 days post-boost infection (see Supplementary Fig. S3). Only minor and no significant changes in the CD8^+^ T cell population were observed (data not shown). Based on these results, we performed histopathological analysis of spleens at 8 days post-primary infection. We observed a noteworthy increase in extramedullary hematopoiesis and in white pulp, mostly attributable to enhanced granulopoiesis as described^12^. Interestingly, lymphatic nodules were also noticeably reduced in numbers and contained fewer cells than controls (Fig. 8b and see Supplementary Table S1).

## Discussion

Knowledge on the mechanisms underlying innate and adaptive immune responses to *S. suis* is still very limited. A better understanding of *S. suis* complex interactions with the immune system in order to generate an effective immune response against this emerging pathogen is urgently needed as the incidence and severity of *S. suis* human infections continue to increase. Based on previous studies reporting *S. suis* ability to lessen optimal DC immunological functions^8^,^9^,^10^,^11^, in the present work, we attempted to further evaluate *S. suis* capacity to modulate the development of adaptive immune responses towards this pathogen. Indeed, this study addresses for the first time the contribution of CD4^+^ T cells in the development of immune functions during *S. suis* serotype 2 infections using *in vivo*, *ex vivo,* and *in vitro* analyses.

In general, T cells seem to be essential for the development of the host adaptive immune response. A preliminary experiment conducted in our laboratory with TCRαβ KO mice showed that mice devoid of functional CD4 and CD8 T cells die significantly more rapidly than control mice, suggesting an important role for T cells during *S. suis* infection (unpublished observations). Moreover, experiments conducted with CD4KO mice in the present study further showed that these mice have transiently elevated bacteremia levels and die significantly more rapidly than control mice during the infection. However, this protective effect of CD4^+^ T cells was lost during infections with higher doses of *S. suis* and at later time points. Divergent effects on lethality using different bacterial doses as a challenge in experimental sepsis has been demonstrated in previous publications using, for example, mice deficient in Toll like receptor-related pathways^13^,^14^. These results suggest that CD4^+^ T cells might play a limited role in the early control of *S. suis* infection, possibly trough activation of innate immune cells and cellular immunity. This prompted us to further characterize CD4^+^ T cell activation status during *S. suis* infection.

During the systemic phase of *S. suis* infection, among other cytokines, high plasma levels of IL-6, TNF-α, IFN-γ, and IL-10 were observed^15^,^16^,^17^. In this study, total spleen cells were shown to secrete TNF-α, IFN-γ, IL-6, IL-10, CCL3, and CXCL9, suggesting an activation of splenic cells during the infection and a polarization towards a Th1 response. IL-10 production can be related to immune regulation and homeostasis^16^, while the presence of CCL3 and CXCL9 suggests that T cells might be recruited in the spleen through the release of these chemokines, as is the case for other streptococci^18^. Evaluation of the production of these cytokines and chemokines through similar experiments conducted in parallel with Group B *Streptococcus* (GBS) has allowed comparison of the levels obtained during *S. suis* and GBS infections. In fact, GBS is often compared to *S. suis*, as both pathogens are encapsulated and cause invasive infection leading to sepsis and meningitis. Furthermore, GBS and *S. suis* are the sole Gram-positive bacteria harbouring terminal sialic acid in their CPSs. However, despite these similarities, the production of TNF-α, IFN-γ, IL-6, IL-10, CCL3, and CXCL9 was much lower in the spleen of *S. suis*-infected mice than it was in GBS-infected mice under the same experimental conditions^19^.

CD4^+^ T cells are expected to be major contributors to cytokine release as they shape the adaptive immune response following the initial innate inflammatory response to systemic bacteria^20^. In the present study, multiple *ex vivo* and *in vivo* analyses of either total splenocytes, CD3^+^ T cells or CD3^+^CD4^+^ T cells suggested that a Th1 response was developed after *S. suis* infection. However, frequency of activated CD4^+^ T cells and levels of IFN-γ, TNF-α, and IL-2 were very low. Production of IL-10 by CD4^+^ T cells activated by *S. suis* was also observed. Besides Treg, IL-10 production has been reported by both Th1 and Th2 differentiated T cells^21^. IL-4 production by CD4^+^ T cells was not detected in our system. The very acute course of the *S. suis* infection might thus suggest that CD4^+^IL-10^+^ cells are generated during the type-1 inflammatory process rather than through the expansion or generation of a particular Treg population.

The development of immune memory was also evaluated. Two weeks after primary infection, surviving animals were challenged with a second infection. Albeit CD4^+^ T cell response was engaged more rapidly (48 h after challenge), levels of cytokine production were similar to those observed after a primary infection. Limited development of a memory response was further supported by the lack of expansion of the effector memory and central memory CD4^+^ T cell subsets in the spleen of *S. suis-*infected mice, even after a boost infection. In contrast, GBS-infected mice have been reported to display enhanced % of central memory CD4^+^ T cells following a boost-infection under the same experimental conditions^19^.

*S. suis* possesses a thick CPS known to be its most important virulence factor. The presence of CPS shelters immunogenic components of the *S. suis* cell wall, impairing host cell activation^8^,^9^,^10^. Previous studies with DCs and macrophages showed that the CPS modulates important cell functions, mostly by interfering with *S. suis* internalization and killing in addition to hampering cell activation^8^,^9^,^10^,^22^. The CPS itself was shown to impair signaling pathways involved in phagocytosis^23^,^24^. In the present study, *S. suis* CPS was found to interfere with the release of IFN-γ, TNF-α, and IL-10 by CD4^+^ T cells. However, the CPS had no effect on the release of IL-2, suggesting that the presence of the CPS interferes with CD4^+^ T cell activation, but not with T cell proliferation. *In vitro* studies demonstrated that some bacterial CPSs, including *Neisseria meningitidis* group C^25^ and *Streptococcus pneumoniae* type 14 or 19F^26^ CPSs, inhibit the maturation and the pro-inflammatory activities of human macrophages and/or DCs and polarize immune responses toward a regulatory profile. *Cryptococcus neoformans* CPS was shown to impair key functions of antigen-presenting cells, including MHC class II expression, and to dampen Th1 responses^27^. Thus, multiple and distinct pathways are used by encapsulated pathogens to lessen the development of the adaptive immune response.

CD69 is the earliest leukocyte maturation marker and is routinely used to evaluate T cell activation^28^. However, it has been reported that CD69-deficient lymphocytes had a normal proliferative response^28^. In the case of *S. suis*, only low expression of CD69 was observed on CD4^+^ T cells activated *in vitro*. This is in contrast to GBS and *S. pneumoniae* which induce a significant up-regulation of CD69 on T cells during the infection^19^,^29^. *S. suis* also failed to induce significant levels of surface expression of CD40L, an important co-stimulatory molecule involved in T cell activation. The CPS does not seem to interfere with CD69 or CD40L expression by *S. suis*-stimulated CD4^+^ T cells. Altogether, these findings suggest that *S. suis* uses multiple virulence factors to reduce either cytokine release or co-stimulatory molecule expression by CD4^+^ T cells. T cell activation is known to depend on DC maturation state: while phenotypically mature DCs can induce T cell proliferation, it appears that only fully activated DCs producing cytokines can effectively activate T cells^4^. Interestingly, *S. suis* is known to modulate DC IL-12 production^8^,^11^, which is a critical signal for effective activation of T cells^30^.

In agreement with an overall impaired activation of T cells, during the infection, we also observed that the production of antibodies against *S. suis* or against a bystander antigen is low. After the infection with *S. suis,* animals developed a bacteremia accompanied by either mild or severe clinical signs such as rough hair coat, swollen eyes, depression, prostration, and weakness. During the primary infection, infected animals displaying severe clinical signs were shown to produce significantly less OVA-specific antibodies compared to animals presenting mild clinical signs. The exact mechanisms responsible for the suppression of the immune response during *S. suis* infection will need further investigations. However, systemic inflammation triggered by pathogens or trauma has already been suggested to lead to prolonged immunosuppression through modulation of DC differentiation and function^30^,^31^. Moreover, it has previously been demonstrated that *S. pneumoniae* inhibits IgG responses to a number of co-immunized soluble antigens. More precisely, *S. pneumoniae* was found to mediate a significant reduction in the formation of Ag-specific splenic T follicular helper and germinal center B cells and antibody-secreting cells in the spleen and bone marrow in response to OVA^32^. Similarly, in a lethal coinfection model of influenza virus and *S. pneumoniae*, coinfection caused depletion of CD4^+^ T cells and B cells in mediastinal lymph nodes and spleen, with concomitant reduction in virus-specific antibody responses^33^. In this regard, a transient depletion of CD4^+^ T cells in the spleen was observed during a primary *S. suis* infection. It is unknown if this is related to cell death or cell emigration to other target tissues or organs. Interestingly, lymphatic nodules were also noticeably reduced in numbers and contained fewer cells than controls, suggesting a possible reduction in T cell-B cell interactions, with consequent reduction in antibody production. Finally, the increase in extramedullary granulopoiesis in the spleen might reflect exhaustion of neutrophil reserves in the bone marrow in response to *S. suis* systemic infection.

To conclude, we demonstrated that CD4^+^ T cells are involved in the development of a Th1 response during *S. suis* infection. However, *S. suis* CPS and possible other virulence factors interfere with CD4^+^ T cell activation, while having no impact on T cell proliferation. The overall reduced CD4^+^ T functional fitness and the lack of significant expansion of memory T cells compromise optimal development of adaptive immune responses, including antibody production. This study is a noteworthy starting point for future research regarding T cell-dependent immunity during *S. suis* infection and its consequences in vaccine development.

## Materials and Methods

### Bacterial strains and growth conditions

The *S. suis* serotype 2 virulent strain P1/7, originally isolated from a case of porcine meningitis, and its isogenic non-encapsulated mutant strain Δ*cpsF* were used. These strains were already characterized and used in previous studies^8^,^34^. *S. suis* strains were grown on sheep blood agar plates and isolated colonies were used as inocula for Todd–Hewitt Broth (THB), which was incubated 8 h at 37 °C with agitation. Working cultures were obtained by inoculating 10 μl of a 10^−3^ dilution of these cultures in 30 ml of THB and incubating for 16 h at 37 °C with agitation. Bacteria were washed twice in phosphate-buffered saline (PBS), pH 7.3, and appropriately diluted in fresh medium to desired concentrations. The number of CFU/ml in the final suspension was determined by plating samples onto THB agar using an Autoplate 4000 Automated Spiral Plater.

### Antibodies

Anti-mouse antibodies used for FACS analysis were as follows: FITC-conjugated anti-CD3 (17A2) and anti-CD4 (GK1.5); PE-conjugated anti-CD4 (GK1.5), anti-CD8 (53-6.7), anti-CD19 (6D5), anti-CD40L (MR1), anti-CD69 (H1.2F3), anti-IFN-γ (XMG1.2), anti-TNF-α (MP6-XT22), anti-IL-2 (JES6-5H4), and anti-IL-10 (JES5-16E3); PE-Cy5-conjugated anti-CD3 (145-2C11); PE-Cy7-conjugated anti-NK-1.1 (PK136) and anti-CD44 (IM7); APC-conjugated anti-IFN-γ (XMG1.2), anti-TNF-α (MP6-XT22), and anti-IL-7Rα (A7R34) and BV421-conjugated anti-CD62L (MEL-14).

### Mice and experimental infections

5 week-old female CD4KO (B6.129S2-*Cd4*^*tm1Mak*^/J) or control C57BL/6 mice (Jackson Laboratory) were used for the mice experiments. All mice procedures were performed in compliance with the guidelines and policies of the Canadian Council on Animal Care and the principles set forth in the Guide for the Care and Use of Laboratory Animals, and protocols were approved by the Animal Welfare Committee of the University of Montreal (Research Protocol Number: Rech-1399). On the day of the experiment, a 1 ml volume of either the bacterial suspension or the vehicle solution (sterile THB) was administrated by intraperitoneal injection (i.p.). Mice were monitored daily to record mortality and clinical signs of disease, such as depression, rough appearance of hair coat, and swollen eyes^15^,^34^. Mice exhibiting extreme lethargy were considered moribund and were humanely euthanized. To determine the level of infection, numbers of viable bacteria in blood were quantified at different times post-infection. Blood (5 μl) was collected from the tail vein, serially diluted in PBS and plated using an Automated Spiral Plater. After overnight incubation, colonies were counted and expressed as CFU/ml.

### Generation of mouse bone marrow-derived dendritic cells

DCs were generated from naive C57BL/6 mice as previously described^8^,^9^. Briefly, bone marrow was removed from femurs and tibiae. After red blood cell lysis, total bone marrow cells (2.5 × 10^5^ cells/ml) were cultured in complete medium consisting of RPMI 1640 supplementd with 5% heat-inactivated fetal bovine serum, 10 mM HEPES, 20 μg/ml gentamycin, 100 U/ml penicillin-streptomycin, 2 mM L-glutamine and 50 μM 2-mercaptoethanol. Complete medium was complemented with 20% GM-CSF from a mouse GM-CSF-transfected cell line (Ag8653) as a source of GM-CSF^35^. Cells were cultured for 7 days and were fed on days 3 and 5. On day 7, clusters were harvested and subcultured overnight to remove adherent cells. Non-adherent cells were collected on day 8, washed, and used as immature DCs for the studies. Cell purity routinely comprised 86–90% CD11c^high^ and F4/80^−/dim^ cells, as determined by FACS analysis and as previously reported^8^,^9^.

### Isolation of splenic CD4^+^ T cells

Untouched CD4^+^ T cells were purified from the spleen of either naive or infected C57BL/6 mice by negative selection using CD4^+^ T cell isolation kit II according to the manufacturer’s instructions (MACS, Miltenyi Biotec). Briefly, spleens were harvested from naive or infected mice at the indicated times (see below) and perfused with RPMI complete medium (without antibiotics), teased apart, and pressed gently through a sterile fine wire mesh. After red blood cell lysis, splenocytes were resuspended in sterile PBS containing 2 mM EDTA and separated using Lympholyte-M density gradient. Low-density cells at the interphase were collected and further purified by magnetic-activated cell sorting (MACS) negative selection as mentioned above. The enriched CD4^+^ T cells had > 95% purity as determined by FACS using anti-CD3 and anti-CD4 staining.

### *In vivo* infection model

In the studies with CD4KO mice, mice were injected i.p. with a single dose of 1 or 5 × 10^7^ CFU of *S. suis* strain P1/7, based on previous work^16^. For the rest of the experiments, C57BL/6 mice were injected with the low dose only (1 × 10^7^ CFU) as ∼50% of animals died with the high dose within the first 48 h post-infection. Surviving animals that had previously displayed clinical symptoms were boosted with a second dose of 1 × 10^7^ CFU of *S. suis* strain P1/7 two weeks after initial infection. Bacteremia was monitored during the first 72 h post-primary infection or the first 24 h post-boost infection. For analysis of CD4^+^ T cell cytokine production, spleens of C57BL/6 mice with clinical symptoms and positive bacteremia were harvested 96 h post-primary infection or 48 h post-boost infection (n = 2 per group × 5 individual experimental infections). Five hours prior to spleen collection, mice were injected i.p. with 200 μg of Brefeldin A, a protein transport inhibitor. Control (placebo) animals were similarly treated. Splenic CD4^+^ T cells were purified as described above, in the presence of Brefeldin A during all the purification steps. The selected time points are based on pre-trial analysis using different post-infection times (data not shown). Purified CD4^+^ T cells were analyzed for cytokine production by IC-FACS (see below).

For analysis of CD4^+^ T memory subpopulations, spleens (n = 3 per group × 2 individual experimental infections) from C57BL/6 mice were harvested at different times post-primary infection (4 and 7 days) and post-boost infection (2 and 7 days). Total splenocytes were analyzed for memory surface markers by multi-parametric FACS analysis (see below).

For quantification of total number of splenic CD3^+^CD4^+^ T cells or CD3^+^CD8^+^ T cells during infection of C57BL/6 mice, spleens (n = 4 per group × 2 individual experimental infections) were collected at different times post-primary infection (2, 4, 6, and 8 days) and post-boost infection (2, 4, 6, and 8 days) and cells quantified by FACS (see below). In selected experiments, for the time point of 8 days post-primary infection, half-spleens were preserved in formalin for histopathological analysis in parallel to FACS analysis (see below).

To measure the *S. suis* specific primary antibody response, sera from infected C57BL/6 mice were collected 14 days after primary infection (n = 10).

### *Ex vivo* analysis of total splenocytes

C57BL/6 mice were injected i.p. with a dose of 5 × 10^7^ CFU of *S. suis* strain P1/7. Control mice were injected with the vehicle solution (sterile THB) (n = 3 per group × 3 individual experimental infections). Spleens were harvested 6 h post-infection. The highest dose was selected for these studies to achieve optimal antigenic stimulation at a short post-infection time in order to capture the early events of antigen presentation and immune cell activation within the spleen environment. After red blood cell lysis and washing, total splenocytes were plated at a concentration of 5 × 10^6^ cells/ml in RPMI complete medium (without antibiotics) in 24-well flat bottom plates, and incubated for 48 h. However, after the initial 6 h of *ex vivo* incubation, gentamycin was added to the culture to control the bacterial load and prevent cell toxicity as reported previously^19^. Total splenocytes from control (placebo) animals were similarly treated. Concanavalin A (ConA, 0.1 μg/ml) was used as positive control. Supernatants were harvested at the indicated time point for cytokine analysis by ELISA. In selected experiments, total splenocytes were incubated *ex vivo* for 14 or 48 h as described above. However, Brefeldin A (3 μg/ml) was added during the last 5 h, and either total splenocytes or CD4^+^ T cells (MACS-isolated from the culture wells) were analyzed by IC-FACS (see below). The above described final culture conditions for *ex vivo* analysis were selected based on multiple pre-trials using different post-infection times (6 and 12 h) combined with 14, 24, 48, and 72 h *ex vivo* incubation times (data not shown).

### *In vitro* DC-T cell coculture model

For the coculture model, 1 × 10^5^ DCs were plated in 48-well flat bottom plates for 1 h at 37 °C with 5% CO_2_. Afterwards, 1 × 10^5^ CFU of either *S. suis* WT strain P1/7 or Δ*cpsF* mutant strain (MOI: 1) were added to the wells for 1 h. Extracellular bacteria were killed using 100 μg/ml of gentamycin and 5 μg/ml of penicillin G as previously described^9^. After 1 h of antibiotic treatment and 3 washing steps, 5 × 10^5^ freshly isolated CD4^+^ T cells from naive mice (T cell: DC ratio of 5: 1) were added to the wells. Cocultures incubated with medium alone served as negative controls. Cocultures treated with either ConA (0.1 μg/ml) or phorbol myristate acetate (PMA, 15 ng/ml) + ionomycine (150 ng/ml) served as positive controls. For FACS analysis of surface marker expression, coculture plates were incubated for 3, 8, 24, and 48 h, prior to cell harvesting and FACS analysis. For T cell cytokine expression, coculture plates were incubated for 48 h, then centrifuged and replenished with fresh medium containing 10 ng/ml of mouse rIL-2. Plates were incubated for 3 days allowing a resting period for activated T cells. After 3 days, T cells were harvested, washed, and seeded into 96 well flat-bottom culture plates coated with 5 μg/ml of anti-mouse-CD3 mAb at a final concentration of 1 × 10^5^ cells/well. These plates were incubated for 48 h prior to supernatant harvesting for ELISA testing. Single cell cultures (either DCs or T cells alone) were also included as controls.

### Cytokine and chemokine quantification by ELISA

Levels of IL-2, IL-4, IL-6, IL-10, TNF-α, IFN-γ, CCL3 (MIP-1α), and CXCL9 (MIG) in cell culture supernatants were measured by sandwich ELISA using pair-matched antibodies from R&D Systems, according to the manufacturer’s recommendations. Twofold dilutions of recombinant murine cytokines were used to generate the standard curves. Sample dilutions giving optical density readings in the linear portion of the appropriate standard curve were used to quantify the levels of each cytokine. Absorbance was measured at 450 nm. The results are from at least three independent ELISA measurements.

### FACS analysis

For multi-parametric IC-FACS of *ex vivo* cultures of total splenocytes, 10^6^ cells were washed and treated for 15 min on ice with FcR-blocking reagent (FcγIII/II Rc Ab) in sorting buffer (PBS-1% fetal bovine serum) prior to surface staining with PE-conjugated anti-CD19, PE-Cy7-conjugated anti-NK-1.1, FITC-conjugated anti-CD3 and/or PE-conjugated CD69 mAbs for 30 min on ice. Cells were then fixed and permeabilized using IC Fixation/Permeabilization eBioscience kit as per the manufacturer’s recommendation. Following fixation and permeabilization, intracellular staining was performed with APC-conjugated anti-IFN-γ or anti-TNF-α mAbs for 45 min at room temperature.

For IC-FACS of MACS-purified CD4^+^ T cells from *in vivo* or *ex vivo* experiments, cells (prepared as described above) were stained for 20 min at room temperature with PE-conjugated mAbs directed against the following intracellular molecules: IFN-γ, TNF-α, IL-2, or IL-10.

For multi-parametric FACS analysis of CD4^+^ T memory subpopulations, cells were surface stained with FITC-conjugated anti-CD3, PE-conjugated anti-CD4, PE-Cy7-conjugated anti-CD44, BV421-conjugated anti-CD62L, and APC-conjugated anti-IL-7Rα^+^ mAbs for 45 min on ice. Cells were gated on CD3^+^CD4^+^ cells, followed by gating CD62L^+^CD44^low^ (naive T cells), CD62L^−^CD44^high^ (effector [memory] T cells), and CD62L^+^CD44^high^ (central memory T cells), as shown in Fig. 5a. Analysis of IL-7Rα expression was used to further identify memory cells (CD44^high^IL-7Rα^+^) within these two subsets as previously described^19^.

For cell surface staining of *in vitro* cocultures, cells were stained with FITC-conjugated anti-CD4 mAb for 30 min on ice followed by washing and staining for 30 min with PE-conjugated anti-CD69 or CD40L mAbs.

For quantification of splenic CD4^+^ T cells during the infection, splenic cells collected at different times post-primary and post-boost infection were stained with PE-Cy5-conjugated anti-CD3 mAb for 30 min on ice followed by washing and staining for 30 min with FITC-conjugated anti-CD4 and PE-conjugated anti-CD8 mAbs.

### Histopathology of spleen sections

Formalin-fixed spleen samples were cut into two, routinely processed and embedded in paraffin. Slides were prepared from 4 μm-thick sections and stained with hematoxylin-eosin-phloxin-saffron. The level of lymphoid depletion (lymphatic nodules), granulopoiesis (increased white pulp), and extramedullary hematopoiesis was evaluated on a 4-grade scale (grade 4 being the most severe).

### ELISA for *S. suis*-specific antibodies

Titers of *S. suis*-specific total Ig and IgG subclasses in mouse sera were determined by ELISA. Polysorp immunoplates were coated with 100 μl/well of *S. suis* strain P1/7 (1 × 10^7^ CFU/ml), allowed to dry for 48 h and fixed with 50 μl/well of methanol for 90 min. Before use, plates were washed three times with PBS containing 0.05% Tween-20, and incubated with 100 μl of serial dilutions of mouse sera for 1 h at room temperature. Bound antibodies were detected by incubation with peroxidase-conjugated goat anti-mouse total Ig [IgG + IgM], IgG1, IgG2b, or IgG2c antibodies for 1 h at room temperature. The plates were developed with TMB substrate and absorbance was measured at 450 nm.

### OVA immunization studies

Mice were infected with *S. suis* WT strain P1/7 (1 × 10^7^ CFU) two days prior to immunization with 10 μg of OVA formulated with 20 μg of CpG ODN 1826 as adjuvant. A boost immunization with the same OVA-CpG ODN formulation was given at day 14 post-primary immunization. Serum levels of OVA-specific total Ig, IgG1, IgG2b, and IgG2c were measured by ELISA at 14 and 21 days post-primary immunization. Briefly, polysorp immuno plates were coated overnight at 4 °C with 100 μl of 50 μg/ml of OVA in PBS. After 2 h-blocking with 1% casein solution in PBS-0.05% Tween-20, serial dilutions of serum samples were added and incubated for 1 h at room temperature. After washing, secondary antibodies specific for total Ig and for each IgG subclass were added as described above.

### Statistical analyses

Cytokine and FACS data are expressed as mean ± SEM and analyzed for significance using Student’s unpaired *t*-test. Serum antibody levels were expressed as endpoint titers, the reciprocal of the highest dilution that yielded the background optical density plus 3 times the standard deviation (OD + 3 SD), and were analyzed for significance using ANOVA analysis. For correlation of OVA-specific antibody levels and clinical signs of *S. suis* disease, the antibody production index was obtained by dividing the antibody levels of infected mice (showing either mild or severe clinical signs) by those of control mice. All analyses were performed using the Sigma Plot System (v.9; Systat Software). A *P* < 0.05 was considered as statistically significant.

## Additional Information

**How to cite this article**: Lecours, M.-P. *et al*. Immune-responsiveness of CD4^+^ T cells during *Streptococcus suis* serotype 2 infection. *Sci. Rep.* **6**, 38061; doi: 10.1038/srep38061 (2016).

**Publisher's note:** Springer Nature remains neutral with regard to jurisdictional claims in published maps and institutional affiliations.

## Supplementary Material

Supplementary Information
