# Supplementary material for: Immune-responsiveness of CD4+ T cells during Streptococcus suis serotype 2 infection
Source: Sci Rep. 2016 Dec 1;6:38061. doi: 10.1038/srep38061 (PMC5131321; doi:10.1038/srep38061)
Supplement: Supplementary Information [file srep38061-s1.pdf]

Immune-responsiveness of CD4$^+$ T cells during *Streptococcus suis* serotype 2 infection

Marie-Pier Lecours[1,4], Corinne Letendre[1], Damian Clarke[1], Paul Lemire[1], Tristan Galbas[2], Marie-Odile Benoit-Biancamano[3], Jacques Thibodeau[2], Marcelo Gottschalk[4] & Mariela Segura[1*]

[1]Laboratory of Immunology, [3]Pathology Division and [4]Laboratory of *Streptococcus suis*, Faculty of veterinary medicine, University of Montreal, St-Hyacinthe, Quebec, Canada. [2]Laboratory of Molecular Immunology, Department of Microbiology, Infectiology and Immunology, University of Montreal, Montreal, Quebec, Canada.

*Correspondence and requests for materials should be addressed to M.S. (email: mariela.segura@umontreal.ca)

Supplementary data

Supplementary Table S1. Splenic histopathological scores in naive and infected mice.

	% $CD3^+CD4^+$	Lymphoid depletion	Extramedullary hematopoiesis	Granulopoiesis
Control	17	-	-	-
	18	-	-	-
	17	-	-	-
	18	-	-	-
	17	-	-	-
S. suis	9	2	3	3
	6	2	4	2
	8	2	3	4
	10	2	-	3
	10	1	1	1

Supplementary Table S1. Splenic histopathological scores in naive and infected C57BL/6 mice. Mice were infected intraperitoneally with a dose of 1 x 10^7 CFU of *S. suis* wild-type strain P1/7. At 8 days post-primary infection, % of $CD3^+CD4^+$ T cells were evaluated by FACS from the spleens of infected and control animals. In parallel to FACS analysis, half-spleens were preserved in formalin for histopathological analysis and levels of lymphoid depletion (lymphatic nodules), granulopoiesis (increased white pulp), and extramedullary hematopoiesis was evaluated on a 4-grade scale (grade 4 being the most severe).

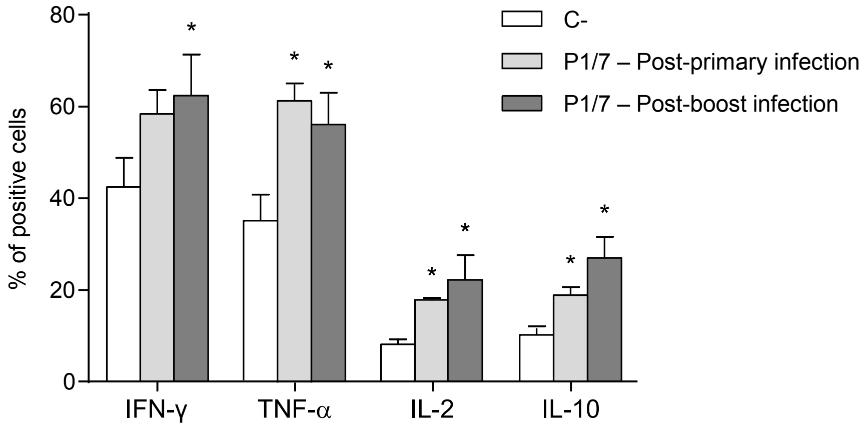

Supplementary Figure S1. **CD4$^+$ T cells produce similar levels of IFN-γ, TNF-α, IL-2, and IL-10 during primary and secondary *S. suis* infections.** Mice were infected intraperitoneally with a dose of 1 x 10^7 CFU of *S. suis* wild-type strain P1/7. Surviving animals who had previously displayed clinical symptoms were boosted with a second dose of 1 x 10^7 CFU of *S. suis* wild-type strain P1/7 two weeks after initial infection. Spleens of animals with clinical symptoms and positive bacteremia were harvested 96 h post-primary infection or 48 h post-boost infection (n = 2 per group x 5 individual experimental infections). Five hours prior to spleen collection, mice were injected with Brefeldin A (200 µg). Non-infected control animals were similarly treated. Splenic CD4$^+$ T cells were MACS-purified, stained intracellularly for different cytokines and analyzed by FACS. Cytokine basal expression levels in non-infected animals were similar at 96 h post-primary mock-infection and 48 h post-secondary mock-infection. Data from the latter time point were selected for the figure (C-). Data are expressed as mean ± SEM (in % of positive cells) from 5 individual experimental infections. FACS was performed using a FACSCalibur instrument. Twenty thousand gated events were acquired per sample and data analysis was performed using CellQuest software. $^*P < 0.05$, indicates statistically significant difference compared to non-infected mice.

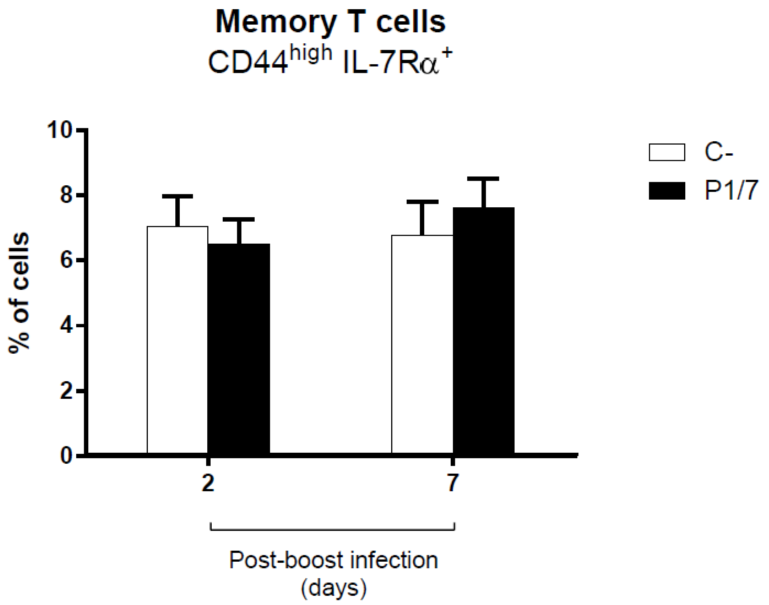

Supplementary Figure S2. Total memory $CD4^{+}$ T cell population remains unchanged in the spleens of *S. suis*-infected mice during a secondary infection. Mice were infected intraperitoneally with a dose of 1 x 10^{7} CFU of *S. suis* wild-type strain P1/7. Surviving animals who had previously displayed clinical symptoms were boosted with a second dose of 1 x 10^{7} CFU of *S. suis* wild-type strain P1/7 two weeks after initial infection. Spleens of animals with clinical symptoms and positive bacteremia were harvested 2 or 7 days post-boost infection. Total splenocytes were stained and analyzed by multi-parametric FACS. Cells were gated on $CD3^{+}CD4^{+}$ cells, followed by gating $CD44^{high}$ cells. The surface marker IL-7Rα was used to further identify memory cells ($CD44^{high}$IL-7Rα^{+}) within this population. Data are expressed as mean ± SEM (n = 3 per group x 2 individual experimental infections). FACS was performed using a FACSCantoII instrument. Thirty thousand events gated on $CD3^{+}CD4^{+}$ cells were acquired per sample and data analysis was performed using Kaluza® Flow Analysis software.

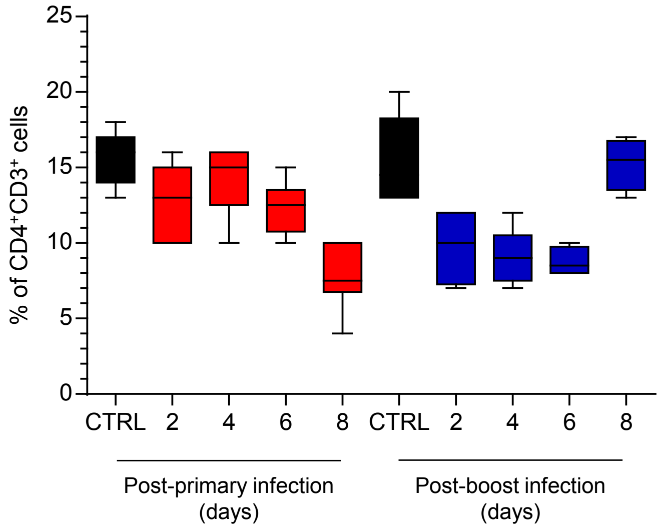

Supplementary Figure S3. Spleens from *S. suis*-infected mice show reduced numbers of $CD4^+$ T which persisted after challenge infection and gradually came back to normal. Mice were infected intraperitoneally with a dose of 1 x 10^7 CFU of *S. suis* wild-type strain P1/7. Surviving animals who had previously displayed clinical symptoms were boosted with a second dose of 1 x 10^7 CFU of *S. suis* wild-type strain P1/7 two weeks after initial infection. Non-infected control animals were included at each time point. Spleens (n = 4 per group) were collected at different times post-primary infection (2, 4, 6, and 8 days) and post-boost infection (2, 4, 6, and 8 days); % of $CD3^+CD4^+$ T cells in the spleens of infected and control animals were evaluated by FACS using a Cell Lab QuantaTM SC MPL MultiPlate Loader instrument. Twenty thousand gated events were acquired per sample and data analysis was performed using Cell Lab Quanta Collection/Analysis software. Quadrants were drawn based on FITC- and PE-Cy5-control stains and were plotted on logarithmic scales. To simplify the graph, "CTRL" represents the pool of non-infected mice throughout the infection period.

Supplementary Results:

***S. suis* induces a weak specific antibody response.** As CD4$^+$ cells play a major role in B cell activation and thus in the generation of a specific humoral response, the anti-*S. suis* antibody response generated during infection was evaluated. Blood from infected mice was collected 14 days after primary infection. As shown in Supplementary Fig. S4, titers of total Ig [IgG+IgM] directed against the whole bacteria were relatively low, when compared to those obtained after an immunization with ovalbumin (OVA) (see Supplementary Fig. S5). Nevertheless, isotype switching was observed in infected animals. In agreement with a Th1 profile, levels of *S. suis*-specific antibodies of the type 1 IgG subclasses (IgG2b and IgG2c) were higher than those of the type 2 IgG1 subclass (Supplementary Fig. S4).

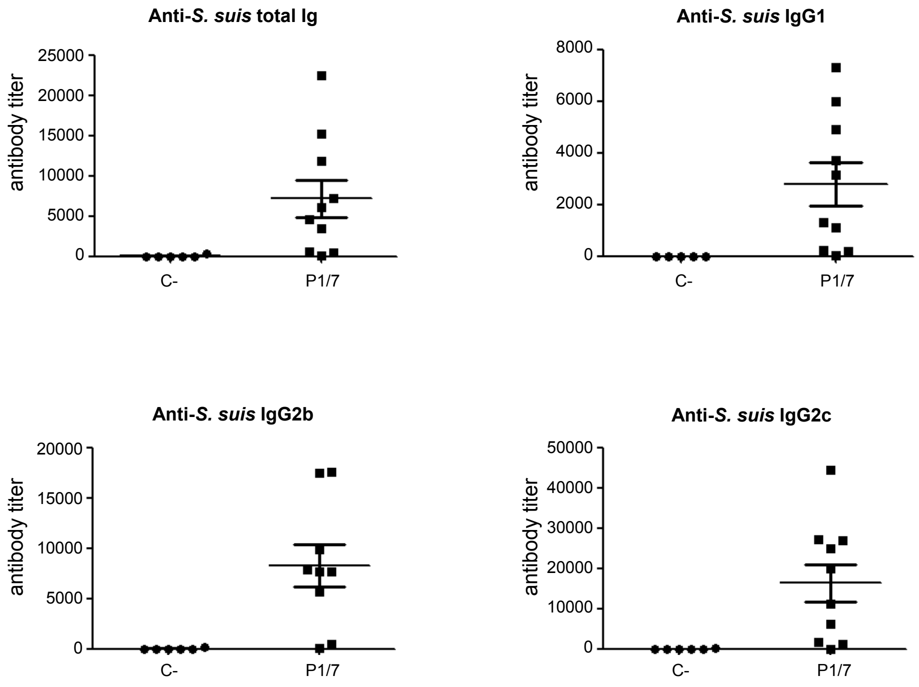

Supplementary Figure S4. *S. suis* induces low serum levels of specific antibodies during a primary infection. Mice (n = 10) were infected with a dose of 1 x 10^7 CFU of *S. suis* wild-type strain P1/7 and sera collected two weeks post-infection. Total Ig [IgG+IgM], IgG1, IgG2b, and IgG2c anti-*S. suis* titers were determined by ELISA. C- represents a pool of control mice (n = 6) injected with vehicle solution.

***S. suis* interferes with the OVA-specific antibody response *in vivo*.** To better understand the overall low antibody response generated during *S. suis* infection, the capacity of *S. suis* to interfere with the development of the antibody response against a bystander antigen was evaluated. C57BL/6 mice were infected with *S. suis* WT strain P1/7 two days prior to the injection of OVA formulated with CpG ODN. Two weeks after primary OVA immunization, serum levels of OVA-specific total Ig, IgG1, IgG2b, and IgG2c were found significantly lower following mice infection with *S. suis* compared to non-infected mice (Supplementary Fig. S5). Surviving mice were then boosted with a second dose of OVA to evaluate the effect of *S. suis* infection on the development of anti-OVA memory antibody response. A significantly lower production of anti-OVA total Ig, IgG1, and IgG2b was also observed after boost. Levels of IgG2c were also reduced after boost in infected mice; however this difference was not statistically significant compared to control mice. Furthermore, during a *S. suis* infection, some animals display severe clinical signs while others present milder symptoms in spite of similar bacteremia levels. A correlation analysis was thus performed between the presence of severe clinical signs in infected animals and the production of OVA-specific antibodies during the primary response. Effectively, as shown in Supplementary Fig. S6, the anti-OVA antibody production was significantly lower in infected animals displaying severe clinical signs than in infected animals showing milder clinical signs.

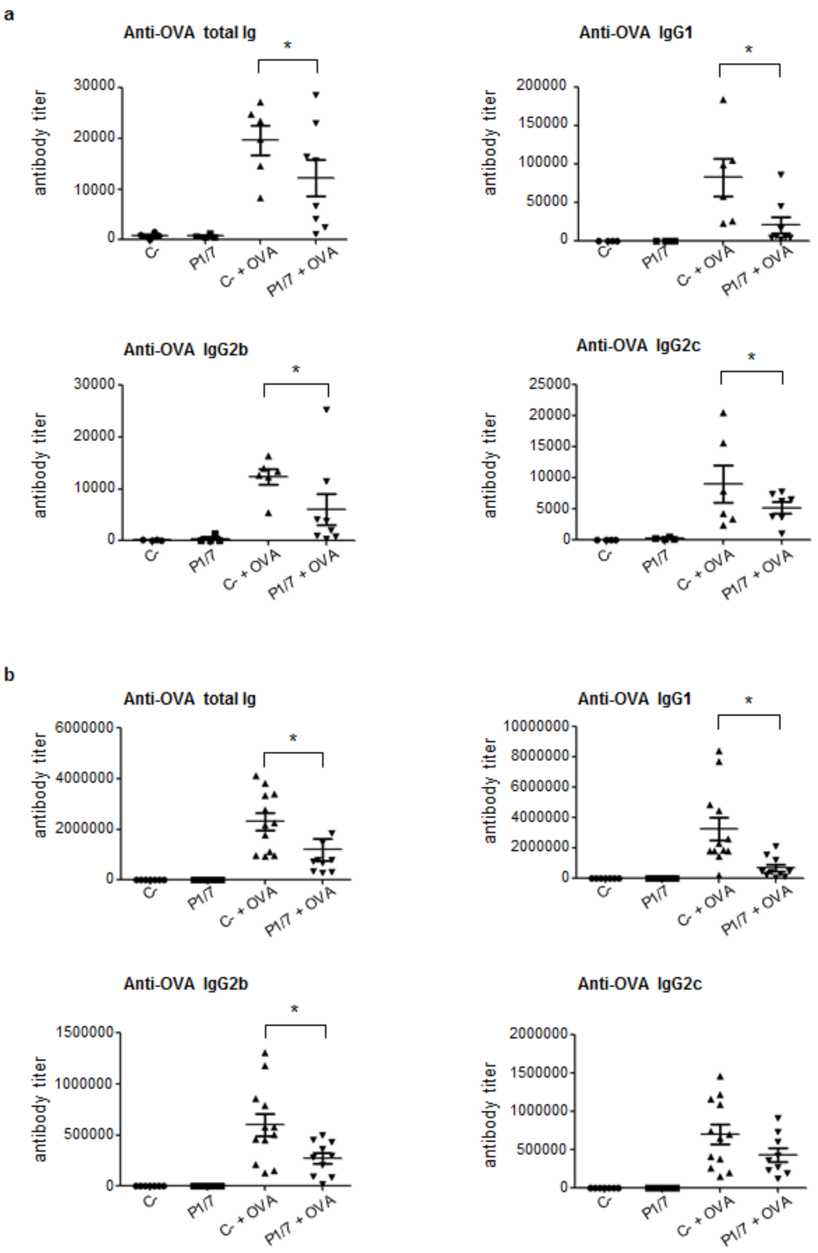

Supplementary Figure S5. The production of ovalbumin (OVA)-specific antibodies is reduced in *S. suis* pre-infected mice. Mice (n = 10) were infected with a dose of 1 x 10^7 CFU of *S. suis* wild-type strain P1/7 two days prior to immunization with 10 µg of OVA formulated with 20 µg of CpG ODN as adjuvant. A boost immunization with the same OVA-CpG ODN formulation was given at day 14 post-primary immunization. Serum levels of OVA-specific total Ig [IgG+IgM], IgG1, IgG2b, and IgG2c were measured by ELISA at (**A**) 14 days and (**B**) 21 days post-primary immunization. C- represents control mice injected with vehicle solution only (n = 6). C- + OVA represents control mice injected with vehicle solution followed by OVA immunization under the same protocol as described above (n = 10). A control group infected with *S. suis* wild-type strain P1/7 only was also included (n = 10). * $P < 0.05$ denotes values that are significantly lower in pre-infected animals (P1/7 + OVA) compared to the non-infected, immunized animals (C- + OVA).

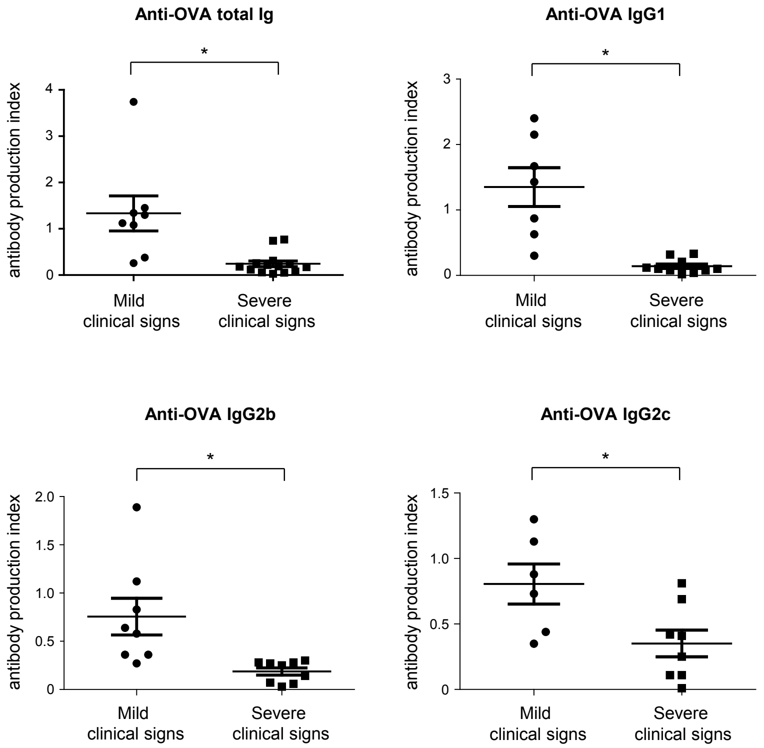

Supplementary Figure S6. Serum levels of OVA-specific antibodies correlate with clinical signs developed by *S. suis*-infected mice. Mice (n = 10) were infected with a dose of 1 x 10^7 CFU of *S. suis* wild-type strain P1/7 two days prior to immunization with 10 µg of OVA formulated with 20 µg of CpG ODN as adjuvant. Serum levels of OVA-specific total Ig [IgG+IgM], IgG1, IgG2b, and IgG2c were measured by ELISA at 14 days post-primary immunization. The antibody production index was obtained by dividing the antibody levels of infected mice (showing either mild or severe clinical signs) by those of control mice. * $P < 0.05$ denotes values that are significantly lower in infected animals presenting severe clinical signs compared to infected animals presenting mild clinical signs.
